# Meta-analysis of the effects of 1-methylcyclopropene (1-MCP) treatment on climacteric fruit ripening

**DOI:** 10.1038/s41438-020-00405-x

**Published:** 2020-12-03

**Authors:** Jing Zhang, Yuanchun Ma, Chao Dong, Leon A. Terry, Christopher B. Watkins, Zhifang Yu, Zong-Ming (Max) Cheng

**Affiliations:** 1 College of Horticulture, Nanjing Agricultural University, Nanjing, China; 2 Plant Science Laboratory, Cranfield University, Bedfordshire, UK; 3 School of Integrative of Plant Science, College of Agriculture and Plant Sciences, Cornell University, Ithaca, NY USA; 4 College of Food Science and Technology, Nanjing Agricultural University, Nanjing, China; 5 Department of Plant Sciences, University of Tennessee, Knoxville, TN 37996 USA; 6 Present Address: Shanghai Key Laboratory of Protected Horticultural Technology, Forestry and Fruit Tree Research Institute, Shanghai Academy of Agricultural Sciences, Shanghai, China

**Keywords:** Plant sciences, Physiology

## Abstract

1-Methylcyclopropene (1-MCP) is an inhibitor of ethylene perception that is widely used to maintain the quality of several climacteric fruits during storage. A large body of literature now exists on the effects of 1-MCP on climacteric fruit ripening for different species and environmental conditions, presenting an opportunity to use meta-analysis to systematically dissect these effects. We classified 44 ripening indicators of climacteric fruits into five categories: physiology and biochemistry, quality, enzyme activity, color, and volatiles. Meta-analysis showed that 1-MCP treatment reduced 20 of the 44 indicators by a minimum of 22% and increased 6 indicators by at least 20%. These effects were associated with positive effects on delaying ripening and maintaining quality. Of the seven moderating variables, species, 1-MCP concentration, storage temperature and time had substantial impacts on the responses of fruit to 1-MCP treatment. Fruits from different species varied in their responses to 1-MCP, with the most pronounced responses observed in rosaceous fruits, especially apple, European pear fruits, and tropical fruits. The effect of gaseous 1-MCP was optimal at 1 μl/l, with a treatment time of 12–24 h, when the storage temperature was 0 °C for temperate fruits or 20 °C for tropical fruits, and when the shelf temperature was 20 °C, reflecting the majority of experimental approaches. These findings will help improve the efficacy of 1-MCP application during the storage of climacteric fruits, reduce fruit quality losses and increase commercial value.

## Introduction

Fruits are stored to maintain fresh quality and extend their shelf life, thereby reducing the loss of commercial value associated with high metabolic rates and disease susceptibility. Fruits can be divided into “climacteric” and “nonclimacteric” types according to respiration patterns during ripening. Increased respiration in climacteric fruits is typically associated with autocatalytic production of the plant growth regulator ethylene, which mediates many aspects of ripening^1^.

1-Methylcyclopropene (1-MCP) inhibits ethylene perception by binding to ethylene receptors to form an ethylene-receptor complex, resulting in delayed fruit ripening^2–4^. 1-MCP was patented in 1996, followed by rapid registration and commercialization because of its nontoxic mode of action, effectiveness at low concentrations, and easy application as a gas. It has also been used extensively as a research tool to study the effects of ethylene on a range of climacteric and nonclimacteric fruits, vegetables, and flowers^5,6^. The impact of 1-MCP on the physiological and biochemical processes related to fruit ripening and consequent effects on quality during storage and shelf life periods can vary greatly among different types of fruits, storage durations and storage conditions^6–8^. The majority of commercial use of 1-MCP is on apples, with less use on other products, in part because it is harder to delay rather than totally inhibit ripening of fruits such as avocado, banana, pear, and tomato.

Specific physiological and biochemical parameters can be used as indicators to assess the degree of response of climacteric fruits to 1-MCP. However, studies with 1-MCP involve different species, concentrations, experimental conditions, and storage conditions. Thus, an integrated, unbiased comparative analysis of the effects of all experimental conditional variables from many studies would be helpful in understanding the effects of 1-MCP on climacteric fruits.

Meta-analysis can be used to integrate the results of individual studies and assess the commonalities^9–11^; its purpose is to objectively understand the results of each study in all relevant contexts of a research topic. Systematically collection of data from each study, along with analysis of publication bias and sensitivity, can verify the stability of the average treatment effect (also known as the “summary effect” of the treatment). The objectives of this study were to carry out a meta-analysis of the effects of 1-MCP treatment on climacteric fruit ripening and thereby determine the effects of different moderating variables on its effects. This study set out to answer the following questions: What is the collective impact of 1-MCP treatments on climacteric fruits? How do specific experimental variables affect the efficacy of 1-MCP treatment on the ripening of climacteric fruits? Which indicators of ripening are most affected by 1-MCP? We then identified further research that is needed to improve the efficacy of 1-MCP in the postharvest storage of climacteric fruits.

## Results

### Publication bias

Studies with relatively high effects are more likely to be published and included in meta-analysis than studies with low effects, which may lead to incomplete studies and publication bias^12^. Several statistical methods of testing for potential bias involve exploring the relationship between study effect size and precision^10^. The idea is that the effect sizes of large studies with higher precision will tend to be smaller than those of studies with smaller sample sizes or higher variance. Nearly half of the funnel plots in our study were asymmetric, indicating that publication bias should be considered (Table 1). Twenty of the 46 summary effects had a Kendall tau value of less than 0.2 and *p* > 0.05, indicating that there was little attention to bias (no tendency for effect sizes to increase as study size decreased). The remaining 26 summary effects had a *p* ≤ 0.05, suggesting publication bias. Egger’s two-tailed significance test (Egger’s *p* value) showed that 26 summary effects may be biased. The results of the trim and fill method showed that 23 summary effects needed to be adjusted for potential deviations. The adjusted value of 18 of these effects was farther from zero than the original value, and that of the remaining 5 summary effects was closer to zero. Therefore, a bias effect was assumed, but it was not expected to affect the main conclusions. As shown in Table 1, all four statistical methods showed that catalase activity and flesh color luminosity may have significant bias, so these summary effects were not included in the meta-analysis.

**Table 1:** Measures used in characterizing publication bias for each effect size

Effect size	Summary effect^a^			Funnel^b^	Kendall^c^		Egger’s^d^		Duval & Tweedie^e^	
	N	ln*R*	*p*	plot	tau	*p*	*β*	*p*	adjusted	#trim
Ethylene production rate	3391	−1.053	0.000	Yes	−0.10	0.00	−0.25	0.01	−1.203	236
Internal ethylene concentration	748	−2.202	0.000	Maybe	−0.01	0.75	−0.69	0.31	−2.720	99
Respiration rate	2722	−0.283	0.000	Yes	−0.08	0.00	−0.12	0.00	−0.283	0
Chlorogenic acid content	58	−0.185	0.005	Maybe	−0.14	0.12	−0.25	0.53	−0.255	8
H_2_O_2_ content	69	−0.060	0.251	No	−0.06	0.46	−0.60	0.03	−0.060	0
MDA content	154	−0.331	0.000	Yes	−0.14	0.01	0.19	0.06	−0.364	29
Flesh ACC content	78	−0.754	0.000	No	−0.08	0.32	−0.40	0.40	−0.878	6
Electrolyte leakage	105	−0.189	0.000	No	−0.25	0.00	−0.06	0.76	−0.239	15
Firmness	4034	0.444	0.000	Maybe	0.14	0.00	0.26	0.00	0.444	0
Pulp firmness	1053	0.321	0.000	Maybe	0.21	0.00	−0.30	0.09	0.321	0
Total soluble solids	1449	0.004	0.603	Maybe	−0.02	0.00	−0.12	0.00	0.004	0
Titratable acidity	1428	0.091	0.000	Yes	−0.03	0.00	0.21	0.00	0.036	386
SSC/TA	267	−0.177	0.000	No	0.05	0.26	0.21	0.01	−0.192	57
Weight loss	821	−0.187	0.000	Yes	−0.04	0.10	−0.11	0.29	−0.187	0
Ascorbic acid content	307	0.131	0.000	No	−0.05	0.90	−0.18	0.00	0.131	0
PG activity	360	−0.244	0.000	Yes	−0.11	0.00	−0.32	0.00	−0.244	0
PME activity	237	−0.328	0.000	Yes	0.05	0.29	0.23	0.03	−0.384	36
LOX activity	94	0.075	0.131	No	0.08	0.24	−0.44	0.05	−0.022	0
β-Galactosidase activity	84	−0.401	0.000	Yes	−0.03	0.67	0.29	0.69	−0.518	14
SOD activity	267	0.077	0.000	No	0.14	0.00	0.11	0.23	0.017	47
POD activity	225	0.109	0.000	Yes	0.09	0.05	−0.14	0.13	0.109	0
CAT activity	216	0.094	0.001	No	0.11	0.02	0.31	0.01	−0.078	76
APX activity	87	−0.001	0.963	No	0.28	0.00	0.34	0.07	−0.092	28
PPO activity	126	−0.208	0.000	No	−0.20	0.00	−0.57	0.01	−0.208	0
Skin ACO activity	70	−0.467	0.001	Maybe	−0.03	0.69	−0.26	0.56	−0.777	20
Flesh ACO activity	78	−0.557	0.000	No	−0.16	0.03	0.34	0.44	−0.650	9
Flesh ACS activity	109	−0.458	0.000	No	−0.14	0.03	−0.10	0.73	−0.665	24
Skin color luminosity	214	0.077	0.000	Yes	−0.05	0.32	−0.24	0.00	0.077	0
Flesh color luminosity	52	0.019	0.442	No	0.41	0.00	0.86	0.00	−0.001	11
Skin color a value	154	−0.014	0.853	No	−0.26	0.00	5.03	0.00	−0.188	61
Skin color b value	55	0.189	0.000	No	−0.23	0.01	−1.73	0.04	0.189	0
Skin color a/b	139	−0.023	0.437	No	−0.12	0.03	−4.93	0.00	−0.119	39
Skin color chroma	251	0.223	0.000	Yes	0.06	0.19	−0.24	0.19	0.223	0
Flesh color chroma	123	0.088	0.001	No	0.11	0.07	0.98	0.00	0.088	0
Skin color hue angle	1412	0.101	0.000	Yes	0.14	0.00	0.23	0.00	0.101	0
Flesh color hue angle	163	0.049	0.027	No	0.22	0.00	0.25	0.00	0.018	40
Chlorophyll content	302	0.434	0.000	Yes	0.08	0.04	0.08	0.72	0.434	0
Lycopene content	229	−0.536	0.000	Yes	−0.14	0.00	−0.19	0.50	−0.618	22
Anthocyanin content	65	−0.571	0.000	No	0.16	0.06	2.19	0.00	−0.754	18
Total carotenoids content	85	−0.452	0.000	No	−0.14	0.05	−0.87	0.01	−0.452	0
Phenolic content	170	0.179	0.000	No	−0.09	0.10	−0.35	0.00	0.179	0
Butyl acetate	52	−2.056	0.000	Maybe	−0.12	0.19	−1.38	0.17	−2.056	0
Ethanol	86	−0.726	0.000	No	−0.12	0.11	−0.87	0.10	−0.858	10
Hexyl acetate	61	−0.947	0.000	No	−0.07	0.44	0.59	0.57	−0.947	0
Total alcohols	64	−1.006	0.000	No	−0.21	0.01	−3.43	0.00	−1.006	0
Total esters	74	−0.886	0.000	No	−0.06	0.44	1.39	0.16	−0.886	0

^a^Summary effect: *n* = number of studies, ln *R* = natural log of the overall summary effect, *p* = probability that the summary effect ≠ 0; ^b^Funnel plot appears asymmetrical; ^c^Begg and Mazumdar Kendall rank correlation: tau = rank correlation coefficient (with continuity correction), two-tailed *p* = probability that the study effect sizes are correlated with their sampling variances; ^d^Egger’s linear regression: *β* = intercept of the regression line, *p* = probability of significant asymmetry in the study effect size/study size association. The regression runs through zero if the funnel plot is symmetrical. The size of the deviation of the intercept from the origin is a measure of asymmetry, with a two-tailed *p* < 0.05 indicating significant asymmetry^12^. ^e^Duval and Tweedie trim and fill: adjusted summary effect after imputing missing studies using an iterative trim and fill procedure, #trim = number of studies imputed in the trim and fill exercise.

### Heterogeneity analysis

**Fig. 1:**
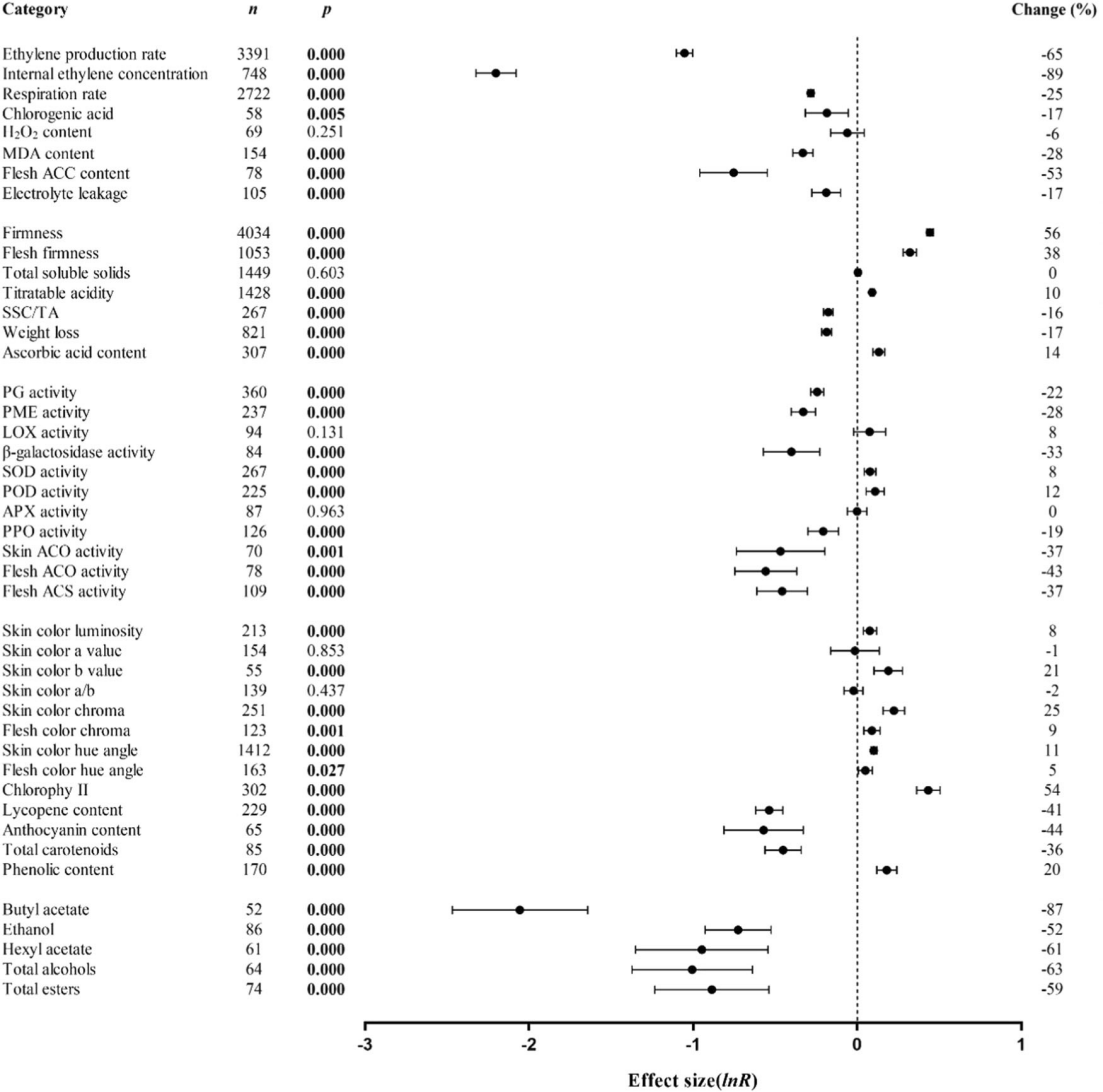
Weighted summary effect sizes (natural log of the 1-MCP-treated/control fruit response ratios, ln R). Horizontal bars associated with summary effects (closed circles) are 95% confidence intervals (CIs). *n* is the number of studies contributing to each summary effect. *p* ≤ 0.05 indicates that the summary effect was significantly different from zero (same for Figs. 2–8 and S1–4)

As shown in Table 2, 23 heterogeneous *p*_*hetero*_ values of the 44 summary effect values in Fig. 1 were significant (*p*_*hetero*_ < 0.100) and had positive I^2^ values, allowing for the use of subgroup analysis to explore the source of true heterogeneity between studies. In meta-analysis, the heterogeneity of the effect size generally refers to the change in the size of the real effect. However, the actual observed changes include (real) heterogeneity and random error, and a *p*_*hetero*_ > 0.1 or *I*^2^ value = 0 does not necessarily mean that true heterogeneity between studies does not exist^9^; substantial real dispersion of real effects may also lead to a *p*_*hetero*_ > 0.1. Therefore, according to the observed pattern, a random effect model was selected to perform the subgroup analysis of the summary effect values for different variables.

**Table 2:** Heterogeneity statistics for the 44 summary effect sizes under 1-MCP treatment

Summary effect size	Qt	*P*_*hetero*_	I^2^	Change (%)
Ethylene production	28877.85	0.0	88.261	**−65**
Internal ethylene concentration	17998.98	0.0	95.850	**−89**
Respiration rate	1904.89	1.0	0.000	**−25**
Chlorogenic acid content	36.99	1.0	0.000	**−17**
H_2_O_2_ content	40.83	1.0	0.000	−6
MDA content	90.54	1.0	0.000	**−28**
Flesh ACC content	338.13	0.0	77.228	**−53**
Electrolyte leakage	43.14	1.0	0.000	**−17**
Firmness	13890.03	0.0	70.965	**56**
Flesh firmness	3816.44	0.0	72.435	**38**
Total soluble solids	294.29	1.0	0.000	0
Titratable acidity	677.00	1.0	0.000	**10**
SSC/TA	72.55	1.0	0.000	**−16**
Weight loss	1950.01	0.0	57.949	**−17**
Ascorbic acid content	50.00	1.0	0.000	**14**
PG activity	184.17	1.0	0.000	**−22**
PME activity	101.26	1.0	0.000	**−28**
LOX activity	97.05	0.4	4.177	8
β-galactosidase activity	38.41	1.0	0.000	**−33**
SOD activity	197.91	1.0	0.000	**8**
POD activity	115.17	1.0	0.000	**12**
APX activity	94.14	0.3	8.643	0
PPO activity	215.22	0.0	41.920	**−19**
Skin ACO activity	259.89	0.0	73.450	**−37**
Flesh ACO activity	265.29	0.0	70.975	**−43**
Flesh ACS activity	273.00	0.0	60.439	**−37**
Skin color luminosity	41.00	1.0	0.000	**8**
Skin color a value	1938.06	0.0	92.106	−1
Skin color b value	59.17	0.3	8.736	**21**
Skin color a/b	226.80	0.0	39.154	−2
Skin color chroma	487.84	0.0	48.753	**25**
Flesh color chroma	150.77	0.0	19.079	**9**
Skin color hue angle	1591.00	0.0	11.314	**11**
Flesh color hue angle	25.39	1.0	0.000	**5**
Chlorophyll content	346.75	0.0	13.194	**54**
Lycopene content	708.66	0.0	67.827	**−41**
Anthocyanin content	179.72	0.0	64.388	**−44**
Total carotenoids content	88.57	0.3	5.155	**−36**
Phenolic content	66.29	1.0	0.000	**20**
Butyl acetate	326.80	0.0	84.394	**−87**
Ethanol	138.52	0.0	38.639	**−52**
Hexyl acetate	420.85	0.0	85.743	**−61**
Total alcohols	372.80	0.0	83.101	**−63**
Total esters	423.01	0.0	82.743	**−59**

Qt, total observed variation among studies; *P*_*hetero*_, probability that Qt was due entirely to sampling error and not to real variation among studies; I^2^, percentage of heterogeneity due to variation among true effects. Bold text signifies that the change was significant (*p* ≤ 0.05), with positive values indicating 1-MCP-induced promotion and negative values indicating 1-MCP-induced inhibition.

### Overall summary effects

Figure 1 summarizes the response ratios of 44 indicators. The objects from 9,344 studies included 33 fruits from 19 plant families, with the most studied group being the Rosaceae (5,128) and the most studied fruit being the apple (2,079). The changes in the various indicators of climacteric fruits and their combined effect values caused by 1-MCP are presented in the form of forest maps.

The inhibitory effect of 1-MCP treatment on internal ethylene concentrations was the most significant, at 89% (Fig. 1). 1-MCP treatment inhibited the other six indicators, including ethylene production and the respiration rate, which were reduced by up to 65%. Treatment did not affect soluble solid concentrations but did affect six quality indicators except for H_2_O_2_ concentrations. Among them, weight loss and soluble solid/acid ratios were reduced by 17% and 16%, respectively. Firmness retention was improved by at least 38%. Titratable acidity and ascorbic acid content were less affected by 1-MCP than the other indicators. Postharvest 1-MCP treatment did not affect the activities of lipoxygenase (LOX) and ascorbate peroxidase (APX) in the fruit. 1-MCP inhibited the activities of enzymes related to cell wall degradation, fruit browning and ethylene biosynthesis, such as polygalacturonase (PG), polyphenol oxidase (PPO), 1-aminocyclopropane-1-carboxylic acid synthase (ACS) and 1-aminocyclopropane-1-carboxylic acid oxidase (ACO) (Fig. 1). 1-MCP treatment also promoted the activities of superoxide dismutase (SOD) and peroxidase (POD). Lycopene, anthocyanin, and carotenoid synthesis were reduced by more than 36%, while the retention of chlorophyll and phenolics was improved by 1-MCP treatment. The accumulation/production of volatiles such as esters and ethanol was reduced by more than 52% in 1-MCP-treated fruit compared with untreated controls.

### Subgroup analysis

From the perspective of biology and production, 16 summary effects, including ethylene production, respiration rate and firmness, were selected for subgroup analysis. The figures of subgroup analysis show the effects of moderating variables (factor) such as species, temperature, and humidity on the summary effect. The effect of 1-MCP on malondialdehyde (MDA) content, electrolyte leakage, PPO, and flesh ACS and ACO activities was little influenced by the moderating variables (Figs. S1–3). However, for each moderating variable, 1-MCP had similar negative effects on lycopene and carotenoid synthesis (Fig. S4). The numbers of studies of these indicators were relatively small, so only the remaining nine indicators were analyzed in detail.

#### Physiological and biochemical indicators

**Fig. 2:**
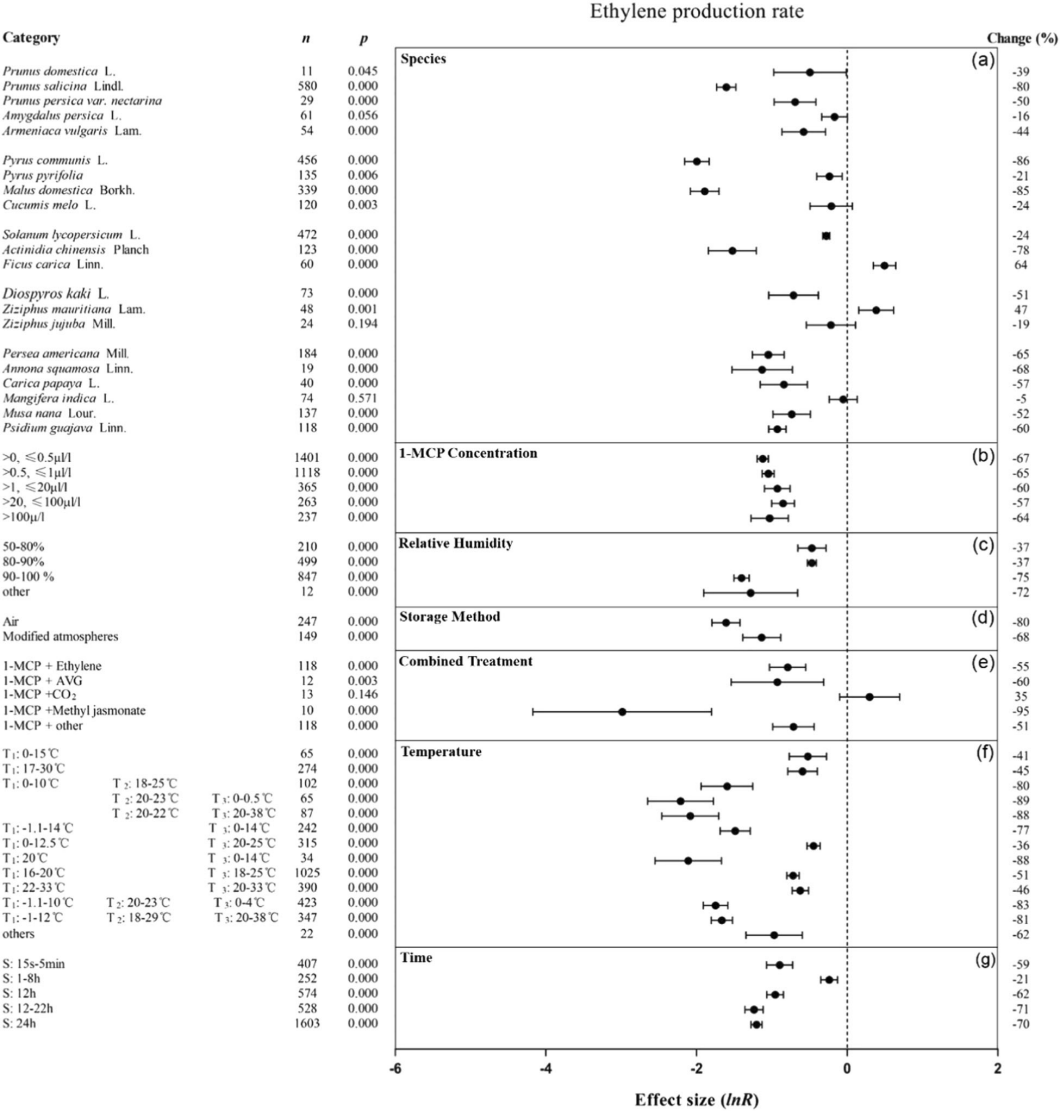
Summary effects (as natural logs, ln *R*) and 95% confidence intervals (CIs) for the effects of 1-MCP treatment on ethylene production. Summary effects were analyzed in fruit exposed to 1-MCP, with the impacts of seven moderator variables on the magnitude of the treatment effect portrayed (**a**–**g**). Category list levels (categories or subgroups) of each moderator. Change (to the right of the plots) refers to the raw percentage increase in ethylene production induced by 1-MCP (T_1_: storage temperature, T_2:_ shelf temperature, T_3_: treatment temperature; S: 1-MCP treatment time (same for Figs. 3–8 and S1–4))

**Fig. 3:**
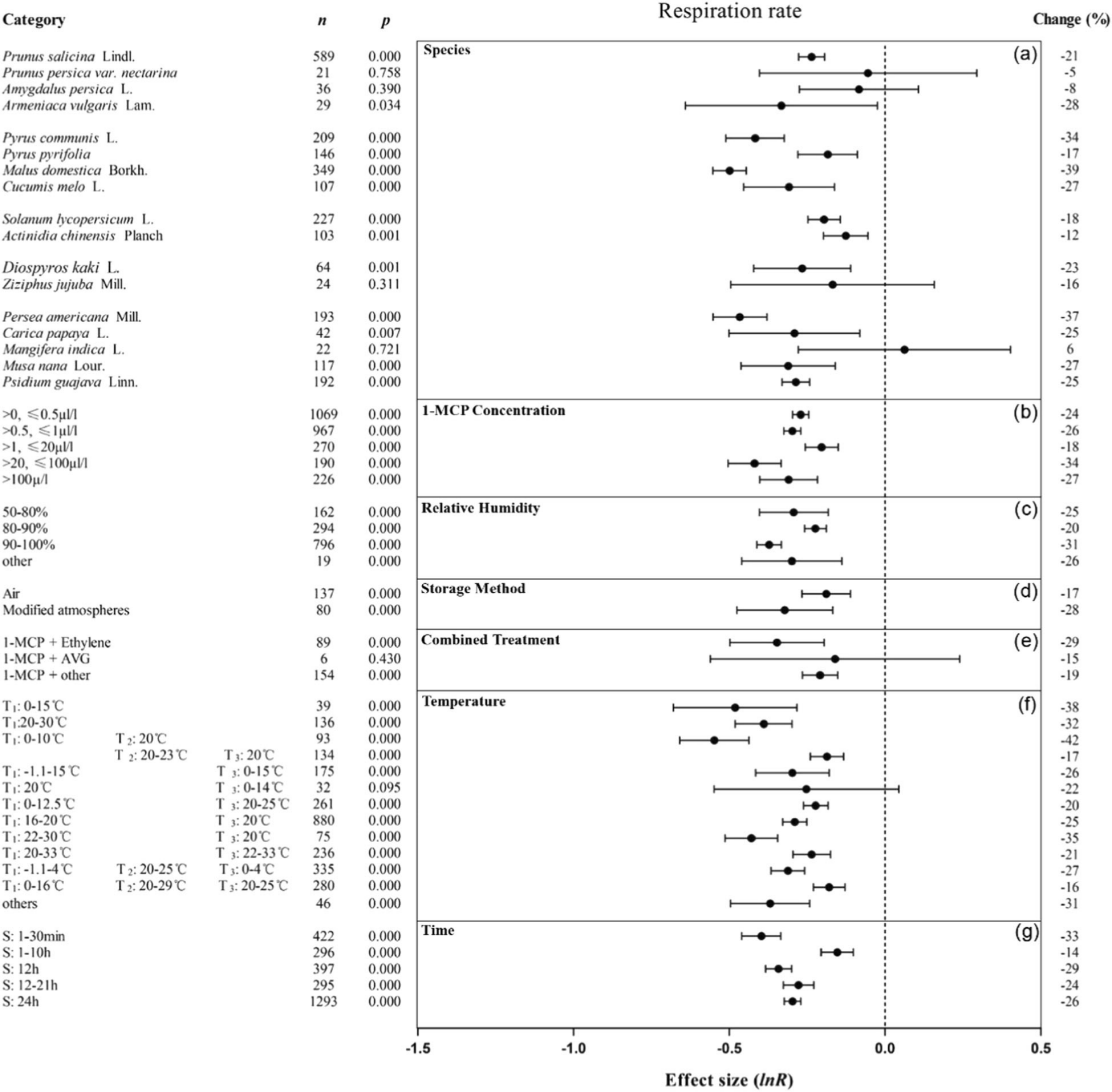
Summary effects (as natural logs, ln *R*) and 95% confidence intervals (CIs) for the influence of 1-MCP treatment on respiration rate. Summary effects were analyzed in fruit exposed to 1-MCP, with the impacts of seven moderator variables on the magnitude of the treatment effect portrayed (**a**–**g**). Category list levels of each moderator. Change refers to the raw percentage increase in respiration rate induced by 1-MCP.

Overall, 1-MCP treatment strongly inhibited physiological and biochemical indicators, ethylene production, respiration rates, MDA content, and electrolyte leakage. Ethylene production was reduced most by 1-MCP treatment (Fig. 2). As shown in Figs. 2a and 3a, 1-MCP decreased the ethylene production and respiration rates in stone fruits (except for peach), pome fruits, and tropical fruits (except for mango). 1-MCP inhibited both the ethylene production and respiration rates of tomato, kiwifruit, and persimmon but increased ethylene production of 1-MCP-treated fig and jujube fruits by up to 64%. Among all types of fruits, 1-MCP had the greatest inhibitory effect on apple and European pear, reducing their ethylene production and respiration rates by at least 85% and 34%, respectively.

The inhibitory effects of 1-MCP treatment on ethylene production, MDA synthesis, and electrolyte leakage were significant and similar in each concentration range (Figs. 2b and S1). The most significant concentration range for 1-MCP inhibition of the respiration rate was 20–100 μl/l (Fig. 3b). 1-MCP inhibited the four indicators most when the relative humidity (RH) was above 90%, with a minimum reduction of 13% (Figs. 2c, 3c and S1). Storage method had little effect on 1-MCP inhibition of ethylene production and respiration rate (Figs. 2d and 3d). Cotreatment of 1-MCP and ethylene inhibited both the ethylene production and respiration rates of the fruits (Figs. 2e and 3e). When the treatment temperature was 0–0.5 °C or 20–38 °C and the shelf temperature was ~20 °C, the inhibitory effect of 1-MCP on ethylene production was enhanced (Fig. 2f). When the storage temperature range was 0–10 °C and the shelf temperature was 20 °C, 1-MCP had a stronger inhibitory effect on the respiration rate (Fig. 3f). The effect of 1-MCP on the inhibition of MDA synthesis and electrolyte leakage was little affected by temperature changes. 1-MCP had a negative effect on the four indicators when the treatment time was greater than 12 h, with a decrease of up to 71% (Figs. 2g, 3g and S1).

#### Quality indicators

**Fig. 4:**
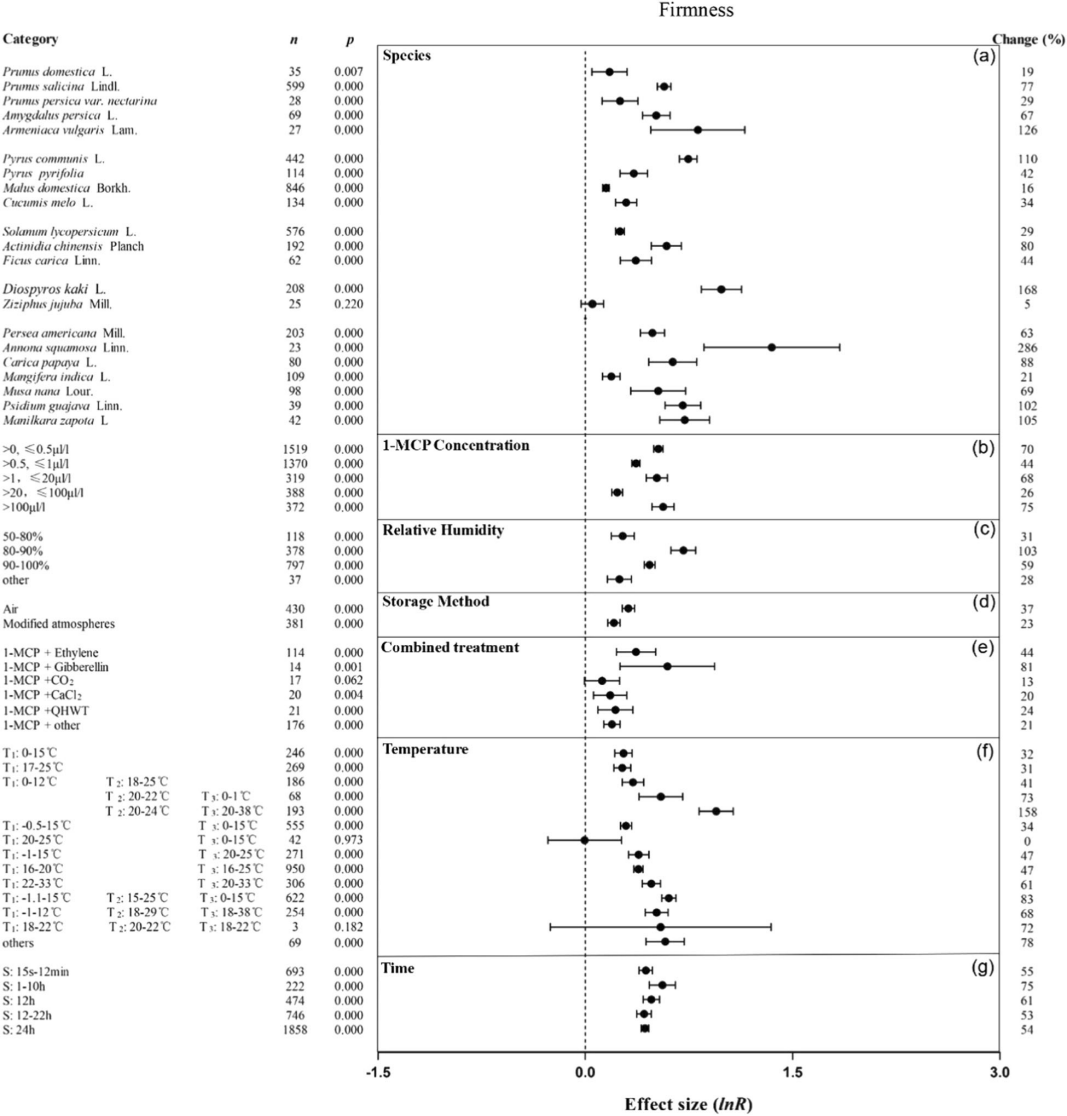
Summary effects (as natural logs, ln *R*) and 95% confidence intervals (CIs) for the influence of 1-MCP treatment on firmness. Summary effects were analyzed in fruit exposed to 1-MCP, with the impacts of seven moderator variables on the magnitude of the treatment effect portrayed (**a**–**g**). Category list levels of each moderator. Change refers to the raw percentage increase in firmness induced by 1-MCP.

**Fig. 5:**
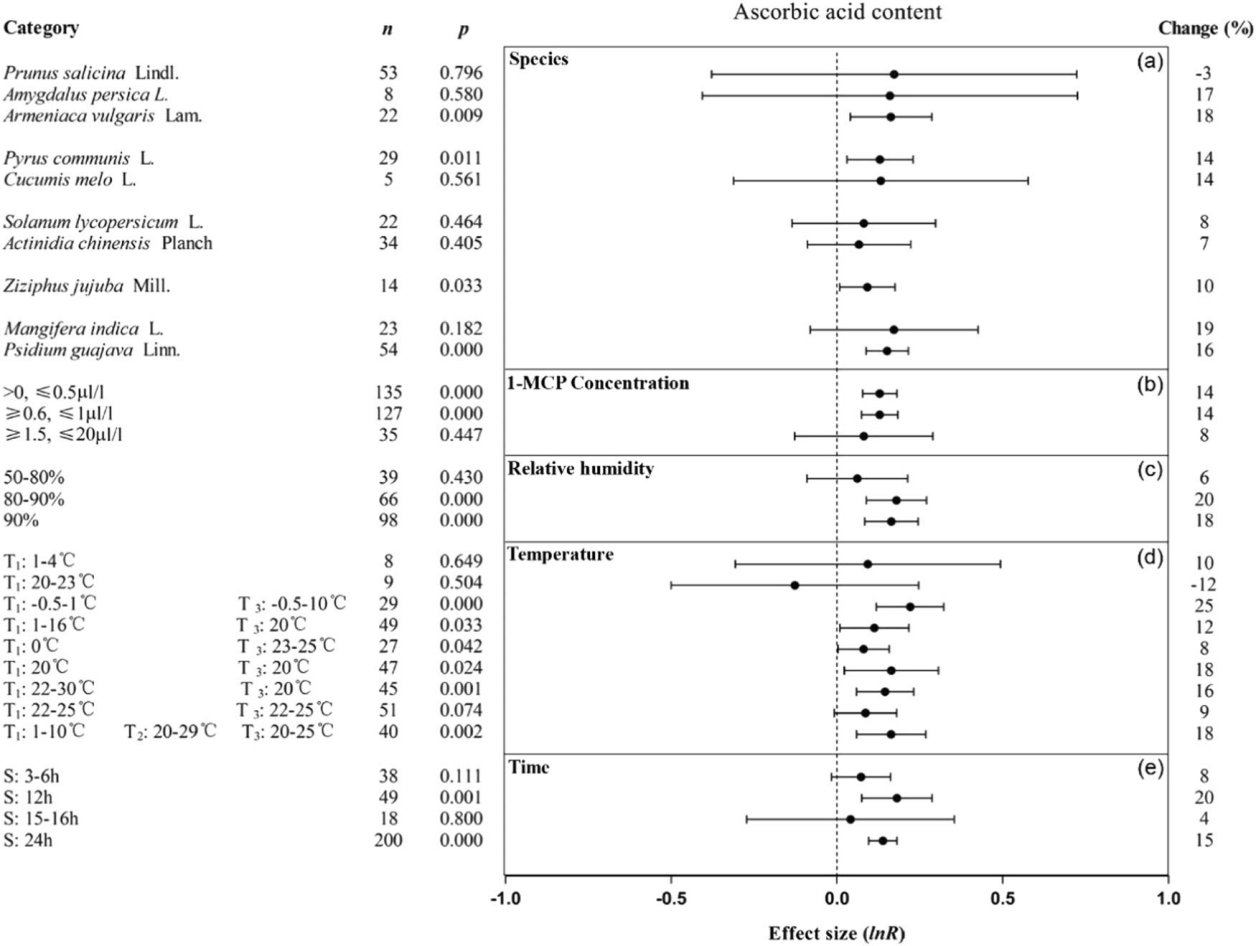
Summary effects (as natural logs, ln *R*) and 95% confidence intervals (CIs) for the influence of 1-MCP treatment on ascorbic acid content. Summary effects were analyzed in fruit exposed to 1-MCP, with the impacts of five moderator variables on the magnitude of the treatment effect portrayed (**a**–**e**). Category list levels of each moderator. Change refers to the raw percentage increase in ascorbic acid induced by 1-MCP.

1-MCP maintained the firmness of stone, pome, berry and tropical fruits (Fig. 4a). Specifically, it maintained the firmness of six fruits, namely, apricot, European pear, persimmon, cherimoya, guava and sapodilla, by at least 102%, but had little effect on jujube fruit when compared with untreated fruit. 1-MCP at every concentration maintained firmness, with the best concentrations range being below 0.5 µl/l, 1–20 µl/l and more than 100 µl/l (Fig. 4b). When the RH was 80–90%, the effects of 1-MCP treatment on maintaining firmness and ascorbic acid were the most significant (Figs. 4c and 5c). Cotreatment of fruit with 1-MCP and ethylene or gibberellin improved firmness retention by 44% and 81%, respectively, which was nearly 2–6 times that of the other treatment groups (Fig. 4e). When the 1-MCP treatment temperature was 20–38 °C and the shelf temperature was 20–24 °C, 1-MCP treatment had the greatest effect on maintaining fruit firmness, which was more than two times that of the other temperature groups (Fig. 4f). The change in treatment time had no effect on the 1-MCP maintenance of fruit firmness (Fig. 4g). The ascorbic acid retention of apricot, European pear, jujube, and guava treated with 1-MCP was improved (Fig. 5a). 1-MCP concentration had little influence on the effect of 1-MCP on maintaining ascorbic acid (Fig. 5b). With the exception of the storage temperature of 20–23 °C, 1-MCP had similar positive effects on maintaining ascorbic acid content among temperature conditions. When the 1-MCP treatment time was 12 or 24 h, the maintenance of fruit ascorbic acid was improved by 20% and 15%, respectively.

#### Enzyme activity indicators

**Fig. 6:**
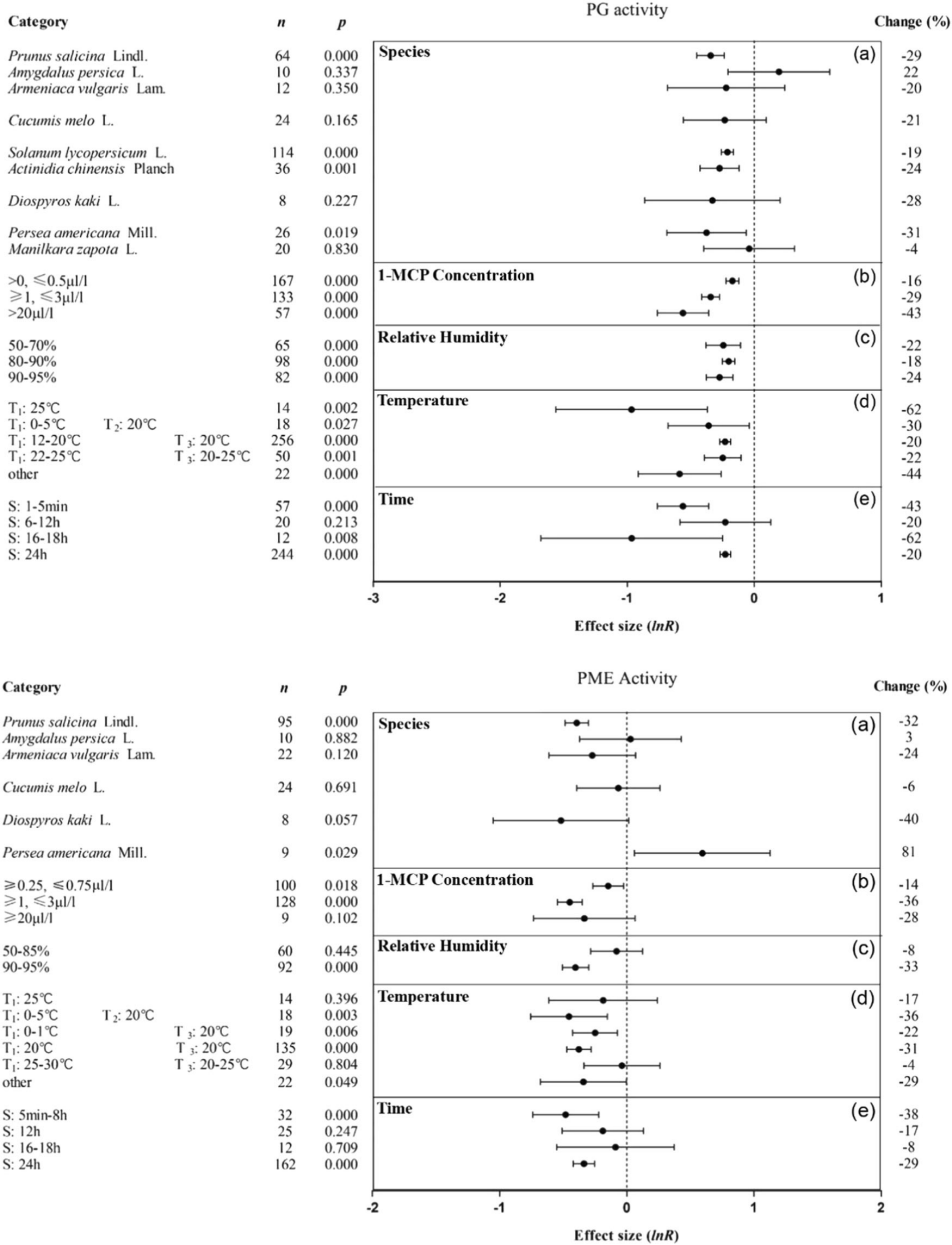
Summary effects (as natural logs, ln *R*) and 95% confidence intervals (CIs) for the influence of 1-MCP treatment on the activity of PG (6-1) and PME (6-2). Summary effects were analyzed in fruit exposed to 1-MCP, with the impacts of five moderator variables on the magnitude of the treatment effect portrayed (**a**–**e**). Category list levels of each moderator. Change refers to the raw percentage increase in activity of PG and PME induced by 1-MCP.

1-MCP inhibited the PG and pectin methylesterase (PME) activities of plum but had little effect on the activities of these enzymes in peach, apricot, melon, and persimmon (Fig. 6-1a, 6-2a). 1-MCP can inhibit the PG activity of tomato, kiwifruit, and avocado (Fig. 6-1a). When the 1-MCP concentration was greater than 20 μl/l, 1-MCP decreased PG activity by 43% (Fig. 6-1b), and 1-MCP had a better effect in inhibiting PME activity at a concentration of 1–3 μl/l (Fig. 6-2b). The optimal concentration of 1-MCP for inhibiting the activities of flesh ACS and ACO ranged from 0.25 to 0.625 μl/l (Fig. S2). When the RH was set at more than 90%, 1-MCP had the strongest effect on inhibiting the activities of PG, PME, and flesh ACS and ACO (Figs. 6-1c, 6-2c, S2). When the storage temperature was 0–5 °C and the shelf temperature was 20 °C, 1-MCP inhibited the activities of PG and PME in fruit (Fig. 6-1d, 6-2d). If 1-MCP treatment was applied for less than 8 h, 1-MCP had the strongest effect on the inhibition of PME activity (Fig. 6-2e). When the treatment time was increased to 16–18 h, 1-MCP inhibited the activities of PG and flesh ACS and ACO (Figs. 6-1e and S2).

**Fig. 7:**
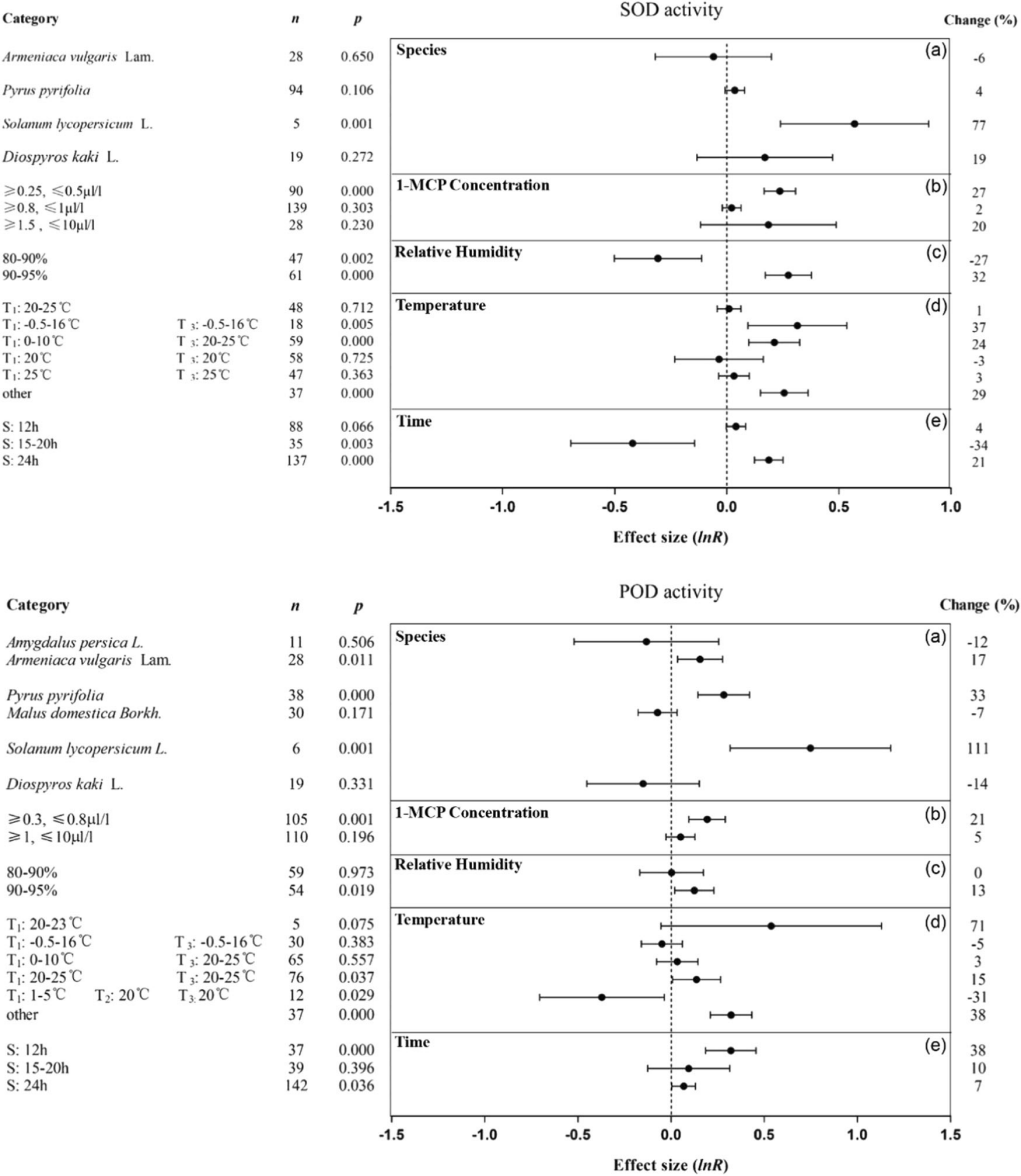
Summary effects (as natural logs, ln R) and 95% confidence intervals (CIs) for the influence of 1-MCP treatment on the activity of SOD (7-1) and POD (7-2). Summary effects were analyzed in fruit exposed to 1-MCP, with the impacts of five moderator variables on the magnitude of the treatment effect portrayed (**a**–**e**). Category list levels of each moderator. Change refers to the raw percentage increase in activity of SOD and POD induced by 1-MCP.

Overall, 1-MCP had a limited effect on promoting SOD and POD activities (Fig. 7). 1-MCP significantly increased the SOD and POD activities of tomatoes by 77% and 111%, respectively (Fig. 7-1a, 7-2a), but had less effect on the activities of these two enzymes in other climacteric fruits. When the concentration was 0.3–0.5 μl/l, the activities of SOD and POD in fruit treated with 1-MCP were increased by at least 21% (Fig. 7-1b, 7-2b). A high RH (90–95%) in the environment improved the effect of 1-MCP on increasing the activities of the two antioxidant enzymes (Fig. 7-1c, 7-2c). The effect of 1-MCP on increasing the activity of SOD was greatest when the treatment and storage temperatures were between −0.5 and 16 °C (Fig. 7-1d). When the treatment time was 24 and 12 h, 1-MCP increased the SOD and POD activities by 21% and 38%, respectively (Fig. 7-1e, 7-2e).

#### Color indicators

**Fig. 8:**
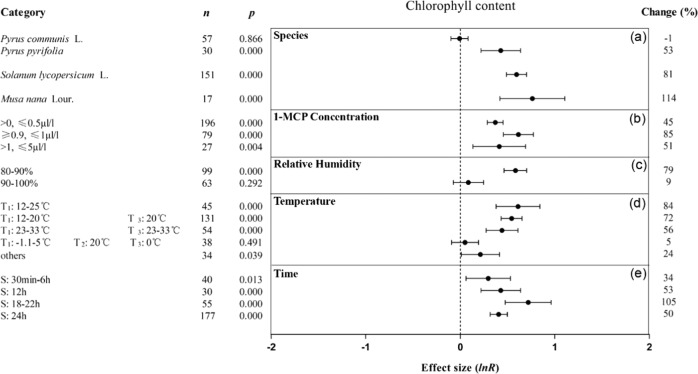
Summary effects (as natural logs, ln *R*) and 95% confidence intervals (CIs) for the influence of 1-MCP treatment on chlorophyll content. Summary effects were analyzed in fruit exposed to 1-MCP, with the impacts of five moderator variables on the magnitude of the treatment effect portrayed (**a**–**e**). Category list levels of each moderator. Change refers to the raw percentage increase in chlorophyll content induced by 1-MCP.

1-MCP treatment more effectively maintained the chlorophyll contents of Asian pears, tomatoes and bananas compared with pears (Fig. 8a) at a concentration of 0.9–1 μl/l (Fig. 8b) and at an 80–90% RH (Fig. 8c). A storage temperature of 12–25 °C improved the ability of 1-MCP treatment to maintain the chlorophyll content (Fig. 8d). When the 1-MCP treatment time was 18–22 h, 1-MCP retained the chlorophyll content more, to nearly 3–5 times that in the other treatment time groups (Fig. 8e).

## Discussion

The application of 1-MCP to climacteric fruits has been studied for decades, but its effects are widely variable and often fruit specific. There are several descriptive reviews on this subject^5–8^, but there have been no systematic, statistics-based assessments of the effect of 1-MCP on horticultural, biochemical, and physiological processes from a global perspective. In this meta-analysis, we focused on the effects of 1-MCP treatment on climacteric fruit ripening, specifically assessing which indicators are most affected by 1-MCP, and analyzed the relationships between factors and impact sizes.

As 1-MCP is an ethylene action inhibitor, its effect can be directly reflected by ethylene production and the activities of the enzymes that are involved in ethylene production (System II). ACS and ACO activities decrease, thereby reducing ACC synthesis and oxidation and decreasing ethylene biosynthesis and, consequently, ethylene-induced respiration. Related research has shown that the formation of volatile compounds and the fruit softening process are dependent on ethylene^13,14^. Therefore, the production of volatile esters in 1-MCP-treated fruit was reduced, which has been reported in some studies, such as studies on banana^15^, peach^16^, and apple^17^. Our meta-analysis showed that 1-MCP treatment inhibited the activities of PME and PG, maintained the integrity of the cell wall to a certain extent, and delayed fruit softening. The accumulation of reactive oxygen species (ROS) in cells can trigger membrane lipid peroxidation, induce membrane rupture, and accelerate the ripening and aging process of fruit^18^. Our meta-analysis shows that 1-MCP can increase the activities of antioxidant enzymes such as SOD and POD, inhibit ascorbic acid degradation, and protect cells from ROS damage. This is reflected in the decreased MDA content and electrolyte leakage rate in 1-MCP-treated fruit. In general, 1-MCP treatment delays the ripening and aging process of climacteric fruits by affecting various physiological processes regulated by ethylene signaling.

The primary ripening behaviors of climacteric fruits, such as softening, color development, and volatile production, are closely related to ethylene production, but species, cultivar, and maturity have an effect on the specific effects of 1-MCP treatment^6,19^. Meta-analysis showed that 1-MCP can inhibit ethylene synthesis and respiration and delay fruit softening and chlorophyll degradation in most climacteric fruits. However, the positive effect of 1-MCP, which delays the degradation of ascorbic acid and cell walls and increases antioxidant enzyme activity, is limited to a few species. This may be due to differences in the sensitivity and dependence of different ripening-related indicators in relation to ethylene, for which Johnston et al.^20^ already found similar patterns in apple-related research^20^. Our study also showed that the effect of 1-MCP in inhibiting ethylene synthesis and respiration was the strongest among Rosaceae fruits (especially apple, European pear) and tropical fruits. However, the effects of 1-MCP treatment are limited in some species. The meta-analysis showed that 1-MCP had little inhibitory effect on the ethylene production and respiration rates of peach, jujube, and mango. Although the low number of compared studies may have led to inaccurate summaries (large CIs), it is still worth noting that 1-MCP may increase the ethylene production of figs and Indian jujube. The reports of Cin et al. and Rasori et al. showed that the effects of 1-MCP on the ripening of peaches are limited, and ethylene biosynthesis is enhanced after treatment^21,22^. In addition, some studies reported that 1-MCP treatment did not affect the ethylene synthesis or respiration rate of fruit but even promoted it, such as in figs^23^, jujube^24,25^ and mango^26,27^. The above research also revealed that the effect of 1-MCP treatment may be closely related to fruit maturity, cultivar, and treatment method. Sisler et al. also proposed that the fruit may produce new ethylene receptors or a low-affinity form of the receptor, resulting in a poor effect of 1-MCP treatment^2^. Exploring the differences in ethylene receptors, 1-MCP diffusivity and metabolism among species is an area worthy of further study.

The response of a specific physiological process to 1-MCP usually depends on the interaction between concentration and exposure time^28^. Inappropriate 1-MCP concentrations and exposure times can result in the final quality of the treated ripe fruit being similar to that of untreated fruit. A 1-MCP concentration of 1 μl/l affects most ripening physiological indicators of climacteric fruits. However, a meta-analysis that included higher 1-MCP concentrations used in aqueous applications showed that when the concentration reached 20 μl/l, the influence of 1-MCP on several indicators further increased. High concentrations of 1-MCP can increase the inhibition of fruit ethylene synthesis and prolong the storage period in tomatoes^29^ and pears^30^. This may provide directions for exploring new commercial 1-MCP treatment concentrations. Meta-analysis shows that when the 1-MCP treatment time is 12–24 h, 1-MCP has a greater impact on various ripening-related indicators than at other durations. Mathooko et al. reported that peach has a weak response to a single treatment with 1-MCP with respect to ethylene biosynthesis and speculated that ethylene receptors can regenerate within a short time based on the expression of ACO and ACS genes^31^. Continuously or intermittently exposing the fruit to 1-MCP to suppress the expression of genes related to ethylene synthesis may be a good method for increasing the effect of 1-MCP.

The storage temperatures of temperate fruits are usually near freezing (0–1 °C), while those of tropical fruits are at 7–15 °C^32^. Our study showed that the optimal treatment or storage temperature was 0 or 20 °C, and a shelf temperature of 20 °C leads to the most effective results of 1-MCP. Commercially, modified atmosphere and low-temperature storage are often used in combination to reduce the overall metabolism of the fruit, delay fruit decay and maintain quality. Storing fruit at low temperatures may cause chilling damage^33^. 1-MCP can suppress disorders related to low temperature, such as superficial scald of apples and pears. However, in fruits such as apricots, peaches, and plums, it can induce disorders such as internal browning, breakdown, and flesh reddening^34^. Brizzolara et al. reported that carbohydrates and amino acids play protective/regulatory roles in chilling injury development^32^, but their involvement in the effect of 1-MCP on these injuries is unclear because the sample size is too small for analysis.

Meta-analysis showed that when the RH was ~90%, 1-MCP treatment was most effective, likely because high humidity can effectively reduce the evaporation of water and dehydration. Water loss of fruit separated from plants is a possible mechanism for promoting ethylene synthesis and maturity^35,36^, as observed in persimmons^37^. Kumar et al. showed that in addition to ethylene, other growth regulators can also fine-tune the fruit ripening process, and the interaction of ethylene and other growth regulators may also affect fruit ripening and postharvest quality^14^. Our meta-analysis shows that the combination of an appropriate growth regulator and 1-MCP may have a positive effect on improving the flavor of the fruit after storage. 1-MCP combined with ethylene, AVG, or GA can delay the ripening of climacteric fruits and improve the quality of fruits after storage, as shown by some studies^38–40^.

Optimization of 1-MCP treatment for fruits of different species has been a research focus since 1-MCP was first discovered, yet its commercial use is still limited. Although its main mechanism of competing for ethylene receptors has been well documented, the variation in effectiveness among fruits^7,8^ suggests that there may be undiscovered mechanisms. In recent years, fewer studies have been centered on improving the commercial value of climacteric fruits during postharvest storage by 1-MCP, while much more research has shifted to the molecular aspects related to the ripening of climacteric fruits after 1-MCP treatment. Transcriptome analysis has been used to reveal ethylene receptor gene expression changes and coexpression changes after 1-MCP treatment^41–43^. However, the relatively low numbers of this type of study make meta-analysis summarizing the molecular mechanisms of 1-MCP in addition to or beyond competing for ethylene receptor unreliable.

Twenty years of studies on the effect of 1-MCP on fruit ripening have generated volumes of data and guidelines for its commercial application. However, our meta-analysis shows that there are still many unanswered questions related to the molecular mechanisms of 1-MCP-induced effects, as well as technology application. For example, when would be the best maturity/ripening stage for each kind of climacteric fruit and cultivar to obtain optimal treatment effects? Can 1-MCP be used flexibly according to the dependence on and sensitivity to ethylene of various indexes at different maturity stages? What is the effect of 1-MCP on the disorders of climacteric fruits during postharvest storage, and what are the relevant physiological mechanisms? How does 1-MCP affect the synthesis and gene expression of ethylene receptors during the ripening of climacteric fruits? How does 1-MCP regulate the physiological processes related to fruit ripening through ethylene signaling pathways, such as aroma volatiles, fruit sugar, and acid conversion? What are the possibilities of combining 1-MCP with growth regulators, such as ABA and GA, or with physical methods such as hot water or intermittent warming treatments to further improve the storage of climacteric fruits? Additional research in these areas is needed to further improve 1-MCP utilization efficiency and the quality of climacteric fruits during storage and reduce the loss of commercial value.

## Materials and methods

The ISI Web of Science system was used to collect the data needed for meta-analysis from 12 electronic databases. Articles were first collected on May 8, 2017, using the search terms “1-methylcyclopropene” and “postharvest physiology” and supplemented with a second search on December 23, 2019. A total of 900 articles were obtained. After further screening, 572 articles were eliminated because they did not meet the inclusion criteria: the study did not involve plants (1 article); the research object was not a climacteric fruit, or the research object was a fresh-cut fruit (207 articles); the research content was not related to postharvest treatment (85 articles); articles were reviews or from books or conferences (179 articles); the document language was not Chinese or English (57 articles); the literature had no data or no control data (5 articles); and the literature was not available (38 articles). In total, 328 articles written in English and Chinese involving 1-MCP treatment of climacteric fruits over 20 years (1999–2019) were selected. Screening with a sample size greater than 45 (from at least two articles) as a statistical criterion resulted in 66 postharvest indicators. Further screening based on whether the indicators were universal and biologically significant resulted in the inclusion of 46 indicators and 292 articles (Supplementary Table provides reference details).

Each study was considered an independent unit for meta-analysis^10,44^. The means and sample sizes for each study were extracted. If no sample capacity or statistical indicators were provided, the sample capacity was defined as *n* = 1 (17 items), and if statistical indicators were reported, it was defined as *n* = 2 (435 items). If the sample capacity was a range, the minimum value was selected (58 items). The data in the chart were extracted using GetData Graph Digitizer (http://getdata-graph-digitizer.com).

Meta-analysis was used to reflect the effects of 1-MCP on various physiological indicators of climacteric fruits (Fig. 1). For each study, the mean of the treatment group relative to that of the control group was chosen as the response ratio for inclusion in the analysis, and its natural logarithm was used for meta-analysis to compare the effect sizes of the treatment^45^:

$${\mathrm{Ln}}\;{R} = {\mathrm{Ln}}\;{Y}_{\mathrm{T}}{/Y}_{\mathrm{C}},$$

where *Y*_T_ and *Y*_C_ are the average values of the experimental and control groups, respectively. The response ratio is often used as a measure of the experimental effect. If the natural logarithmic deviation is small and the sampling approximates a normal distribution, the ratio is an appropriate measurement for meta-analysis^45,46^. A value of Ln *R* greater than 0 indicates an increase in the response of the indicator after 1-MCP treatment, while a value not greater than 0 indicates that the indicator is unchanged or reduced.

The summary effects of ripening-related indicators were calculated and classified into five categories: physiology and biochemistry, quality, enzyme activity, color, and volatiles. Seven moderating quantities used to test for effect size heterogeneity were summarized from various studies, including species, concentration of 1-MCP, RH, storage method (air or modified atmosphere), combined treatment, temperature, and time. Each variable contained at least two levels, with each level containing at least three studies from more than 2 articles. If the classification level did not meet the requirements for meta-analysis, it was classified as “other”.

Comprehensive meta-analysis software (version 2.0, BioStat, Englewood, NJ, USA; 2017) was used for the meta-analysis of the summary effect values. Individual studies were weighted using nonparametric variance methods:

$${\mathrm{VlnR}} = \left( {n}_{\mathrm{T}} + {n}_{\mathrm{C}} \right)/({n}_{\mathrm{T}} \times {n}_{\mathrm{C}}),$$

where *n*_T_ and *n*_C_ are the sample sizes of the experimental and control groups, respectively, and VlnR is the variance of the natural logarithm of the response ratio^9^. When the 95% confidence interval does not include 0 and *p* < 0.05, the summary effect is significant. The Q statistic was used to evaluate whether the effect value was heterogeneous, and *I*^2^ was used to quantify the heterogeneity of the effect size^9,47^. A *p* value of less than 0.1 for the *Q*-test indicates that the summary effect is significantly heterogeneous^48^. An *I*^2^ value of 0 indicates no true heterogeneity, and a larger value indicates a larger proportion of differences observed between studies due to true heterogeneity. Even when articles are collected from multiple sources, some unpublished articles are difficult to obtain, resulting in potential publication bias. In this study, four statistical methods were used to test for publication bias. A funnel plot can intuitively reflect the relationship between the effect size and its standard error^9^. The Begg and Mazumbar rank (Kendall) correlation and Egger’s regression test were used to quantify the bias revealed by the funnel plot^12^. Duval and Tweedie’s trim and fill method was used to assess the potential impact of missing studies and the effect of bias on the results. The results can be divided into three categories: (1) publication bias may not exist, with little effect on the results; (2) publication bias exists but does not affect the main conclusion; and (3) publication bias may affect the main conclusion^9,11,49^. The statistical methods used to assess the asymmetry of the funnel plot were indirect and exploratory^50^, so inferences about the level and impact of potential publication bias are only speculative.

## Supplementary information

Supplementary Figure 1

Supplementary Figure 2

Supplementary Figure 3

Supplementary Figure 4
