# Supplementary material for: Meta-analysis of the effects of 1-methylcyclopropene (1-MCP) treatment on climacteric fruit ripening
Source: Hortic Res. 2020 Dec 3;7:208. doi: 10.1038/s41438-020-00405-x (PMC7713375; doi:10.1038/s41438-020-00405-x)
Supplement: Supplementary file 2 — Supplementary Figure 2 [file 41438_2020_405_MOESM2_ESM.docx]

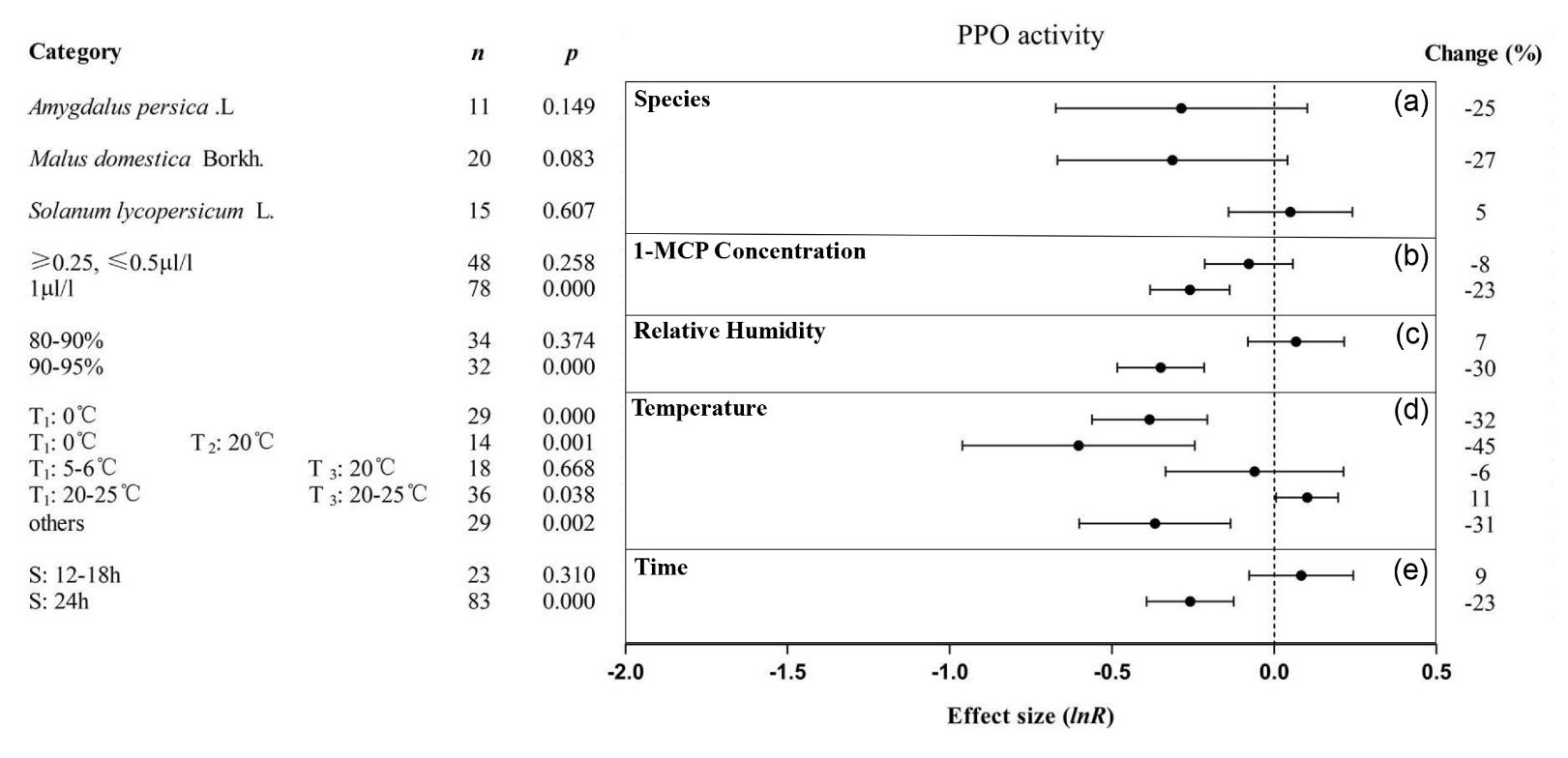


**Fig. S3:** Summary effects (as natural logs, ln R) and 95% confidence intervals (CIs) for the influence of 1-MCP treatment on the activity of PPO. Summary effects were analyzed in fruit exposed to 1-MCP, with the impacts of five moderator variables on the magnitude of the treatment effect portrayed (a–e). Category list levels of each moderator. Change refers to the raw percentage increase in activity of PPO induced by 1-MCP.
