# Supplementary material for: Meta-analysis of the effects of 1-methylcyclopropene (1-MCP) treatment on climacteric fruit ripening
Source: Hortic Res. 2020 Dec 3;7:208. doi: 10.1038/s41438-020-00405-x (PMC7713375; doi:10.1038/s41438-020-00405-x)
Supplement: Supplementary file 4 — Supplementary Figure 4 [file 41438_2020_405_MOESM4_ESM.docx]

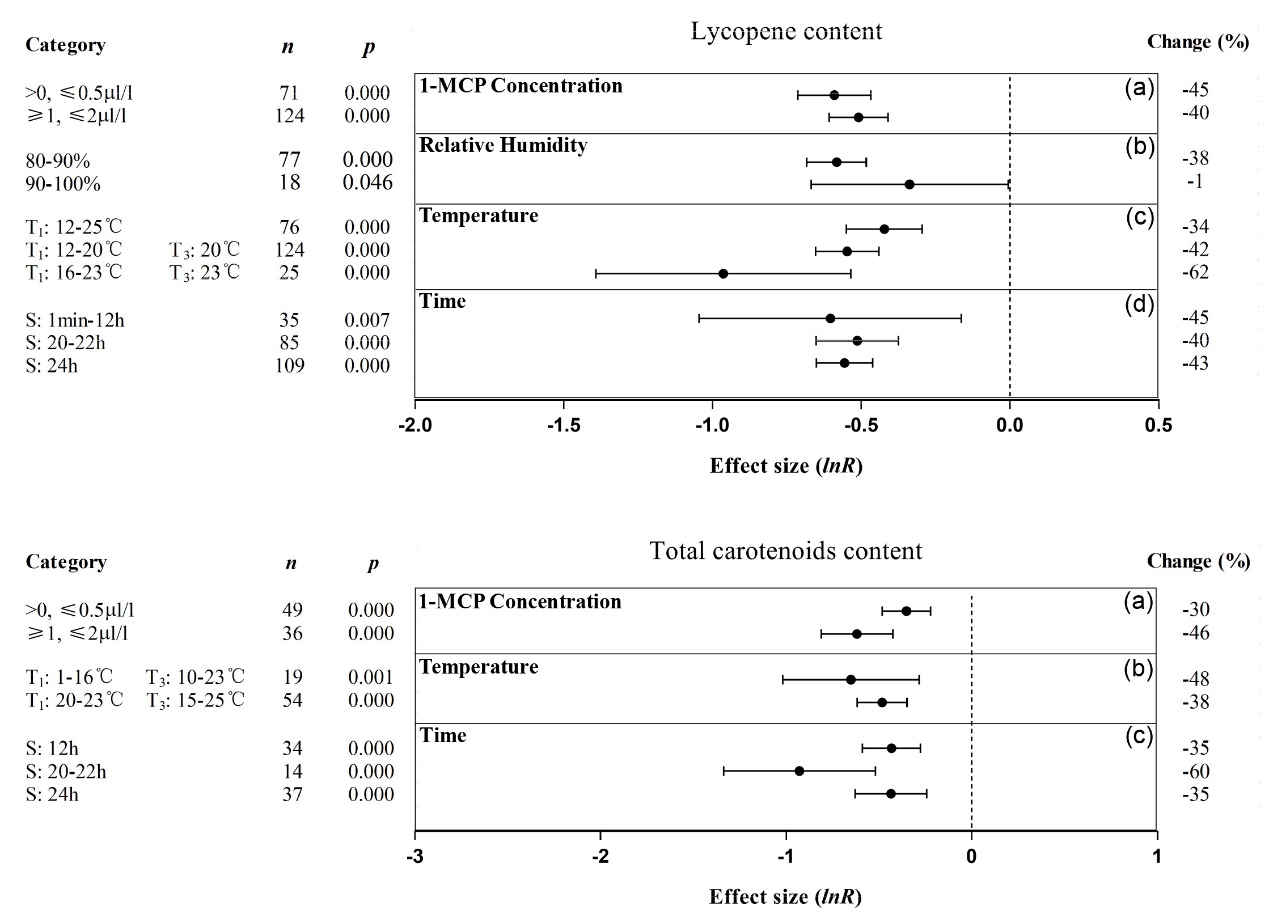


**Fig. S4:** **Summary effects (as natural logs, ln R) and 95% confidence intervals (CIs) for the influence of 1-MCP treatment on the** **content of total carotenoids and lycopene.** Summary effects were analyzed in fruit exposed to 1-MCP, with the impacts of four moderator variables on the magnitude of the treatment effect portrayed (a–d). Category list levels of each moderator. Change refers to the raw percentage increase in content of total carotenoids and lycopene induced by 1-MCP.
